# Interfacial Structure of Carbide-Coated Graphite/Al Composites and Its Effect on Thermal Conductivity and Strength

**DOI:** 10.3390/ma14071721

**Published:** 2021-03-31

**Authors:** Hao Jia, Jianzhong Fan, Yanqiang Liu, Yuehong Zhao, Junhui Nie, Shaohua Wei

**Affiliations:** 1 GRINM Metal Composites Technology Co. Ltd., Beijing 101407, China; chinsland@sina.com (H.J.); liuyanqiang@grinm.com (Y.L.); 18612186108@163.com (Y.Z.); niejunkey@163.com (J.N.); weishaohua666@163.com (S.W.); 2 General Research Institute for Non-Ferrous Metals, Beijing 100088, China; 3 GRIMAT Engineering Institute Co., Ltd., Beijing 101407, China

**Keywords:** Al matrix composites, interfaces, thermal conductivity, carbide coating

## Abstract

Graphite/Al composites had attracted significant attention for thermal management applications due to their excellent thermal properties. However, the improvement of thermal properties was restricted by the insufficient wettability between graphite and Al. In this study, silicon carbide and titanium carbide coatings have been uniformly coated on the graphite by the reactive sputtering method, and Graphite/Al laminate composites were fabricated by a hot isostatic pressing process to investigate the influence on thermal conductivity and mechanical properties. The results show that carbide coating can effectively improve the interfacial thermal conductance of SiC@Graphite/Al and TiC@Graphite/Al composites by 9.8 times and 3.4 times, respectively. After surface modification, the in-plane thermal conductivity (TC) of the composites with different volume fractions are all exceeding the 90% of the predictions. In comparison, SiC is more conducive to improving the thermal conductivity of composite materials, since the thermal conductivity of the 28.7 vol.% SiC@Graphite/Al reached the highest value of 499 W/m·K, while TiC is favorable for improving the mechanical properties. The finding is beneficial to the understanding of carbide coating engineering in the Graphite/Al composites.

## 1. Introduction

With the rapid development of electronic devices, vast quantities of heat caused by integration and miniaturization have become the issue to restrict their reliability and lifetime. Therefore, thermal management materials, such as heat dissipation substrate and heat sink, should have high thermal conductivity (TC), a certain mechanical strength and low thermal expansion coefficient matching with substrate, so as to ensure the thermal stability and avoid device failure due to insufficient heat output [1,2,3]. Nevertheless, some traditional thermal management materials, such as Cu/Mo and Cu/W, could no longer fulfill the requirements for high-power electronic devices [4,5]. Thus, metal matrix composites (MMCs) reinforced with high TC carbon materials, such as carbon fibers [6], graphite, diamond [7], carbon nanotubes [8] and graphene [9], have gradually come into view.

To date, some works have been done as a result of combine graphite with metal composite through different processes. Prieto et al. [10] obtained the graphite flake (Gf)-reinforced composite by infiltrating Al into the preform, which had TC between 294 and 390 W/m·K. Zhou et al. [11] introduced a small percentage of Si particles as spacers between the layers Gf so that metal could be more easily to infiltrated in, which had TC in the range of 179–526 W/m·K. Li et al. [12] manufactured 70 vol.% Gf/Al composites with preferred orientation, and the TC reached to 714 W/m·K. Up until now, existing research still has a big gap in the theoretical value of TC. It is also known that additional amounts of graphite will inevitably lead to a decline in mechanical properties; therefore, it is essential to achieve high TC with a relative low reinforcement volume, so that the composite can still retain good mechanical properties. In addition, the TC of graphite in (002) crystal plane can reach 2200 W/m·K at room temperature [13]. Strong anisotropy on TC means the control of the distribution and orientation of the reinforcements is also a problem, especially for the micron-scaled graphite flake. Based on this, Huang et al. [14,15] used graphite films and aluminum foils as raw materials and fabricated the laminate composites by vacuum hot pressing, successfully solving the problem on orientation: the TC of their obtained composites could reach as high as 902 W/m·K.

Another major problem restricting the TC is the poor wettability of graphite and aluminum, and harmful reaction may occur on the interface. Thus, the strong carbide-forming elements were introduced in order to improve the interfacial bonding strength and hinder the interfacial reaction [16,17].

In this work, two different carbide coatings (SiC/TiC) were applied to the surface of graphite film by reactive sputtering. Then, graphite film and aluminum were fabricated into laminate composites by hot isostatic pressing (HIP), aimed at elucidating the interfacial structure between different carbide coating and Al and to clarify the key factors influencing the TC and mechanical properties of the composites.

## 2. Materials and Methods

### 2.1. Raw Materials

Graphite films and 1060 Al foils (99.6% in purity, with TC of 234 W/m·K) were used as raw materials. Al foils with thickness of 50 μm and 25 μm were purchased from Shanghai Yuanyi Metals Co. Ltd (Shanghai, China) the graphite films with average thickness of 25 μm were acquired from Qingdao Kangboer Graphite Co., Ltd. (Qingdao, China).

**Figure 1:**
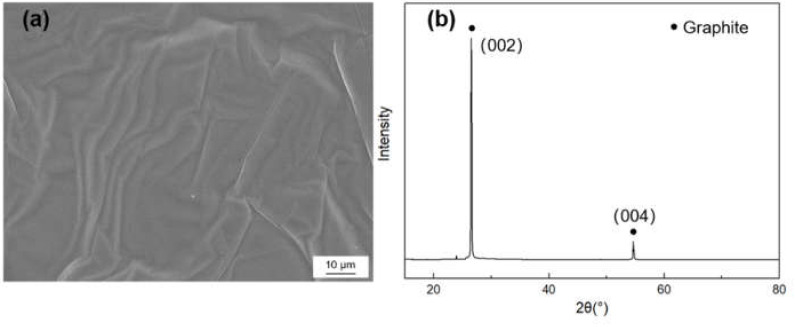
(**a**) SEM morphology and (**b**) X-ray diffraction (XRD) pattern of the graphite film.

Figure 1 shows the morphology and X-ray diffraction (XRD) pattern of the graphite film. The characteristic diffraction peak is (002) peak and (004) peak, in which the graphite’s full width at half maximum (FWHM) and the distance of (002) crystal face can be acquired. The graphitization degree can be calculated by the Mering and Maire formula:

(1) $$g=\frac{0.3440-d_{002}}{0.3440-0.3354}$$

Here, g is the graphitization degree, *d*_002_ is the average layer spacing of crystal face, the layer spacing of the ideal graphite crystal is 0.3354 nm and the layer spacing of amorphous carbon is 0.344 nm. The crystallite parameters of graphite film are listed in Table 1, and the in-plane and axial TC of the graphite films were measured to be 1189 W/m·K and 18 W/m·K, respectively.

### 2.2. Carbide Coating on the Graphite

Graphite films were washed by Ar ions for 20 min to eliminate impurities on the surface. Titanium (purity 99.995%) and silicon (purity 99.99%) were used as the target and controlled by radio frequency cathode. When the background pressure was lower than 3 × 10^−4^ Pa, a mixture of argon and acetylene was introduced into the vacuum chamber, and the total pressure of the mixed gas was kept at 0.5 Pa. In order to improve the adhesion between the film and graphite, a thin titanium (silicon) layer was predeposited on the graphite before reactive sputtering. It was necessary to keep the thickness of carbide coating within 200 nm, due to its low TC. The thickness of the coating layer was controlled by the deposition time.

### 2.3. Preparation of Graphite/Al Composites

The coated graphite films and Al foils were cut into the designed size and subsequently layered into laminate material and heated in a HIP furnace. The volume fractions of graphite were obtained by controlling the thickness of the aluminum foils (11.1%, 18.9% 28.7%). The furnace was heated up to 585 °C by 10 °C/min and held for 120 min in 120 MPa.

### 2.4. Characterization

X-ray diffraction (,XRD-6000, Shimadzu, Kyoto, Japan) was applied to investigate the phase of graphite and composite, and a high resolution transmission electron microscope (TEM, Jem-2100, Jeol, Tokyo, Japan) was used to detect the crystal structure of the films. The microstructure was observed by an optical microscope (OM, Axiovert A1, Zeiss, Oberkochen, Germany) and a field emission scanning electron microscope (FE-SEM, JSM-7600F, Jeol, Tokyo, Japan). Energy dispersion spectra (EDS) attached to the FE-SEM was applied to determine and analyze the elemental composition and distribution. The TC of composite λ was calculated by the following formula:

(2) $$\lambda =\alpha \times C_{p}\times \rho$$

Here, α is the thermal diffusion coefficient of the composite measured at room temperature by a laser flash thermal analyzer (LFA-447, Netzsch, Selb, Germany), ρ is density of the composite measured by Archimedes principle, $C_{p}$ is specific heat of the composite, which was calculated according to rule of mixture:

(3) $$C_{p}=C_{Al}V_{Al}+C_{Gr}V_{Gr}$$

$C_{Al}$,$\;C_{Gr}$ is the specific heat and $V_{Al}$, $V_{Gr}$ is the volume fraction of aluminum and graphite, respectively.

## 3. Results and Discussion

### 3.1. Microstructures Characterization of the Coated Graphite

**Figure 2:**
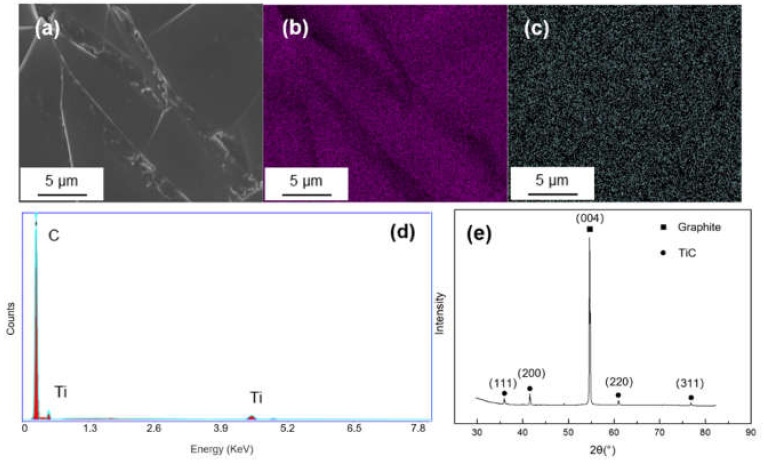
(**a**) SEM morphology and of Ti-coated graphite; (**b**,**c**) EDS mapping results of C and Ti; (**d**,**e**) EDS spectrum and XRD pattern of the Ti-coated graphite.

The surface morphology and energy dispersion spectra (EDS) mapping result of Ti-coated graphite are illustrated in Figure 2. The coating surface was integrated and homogenous, and it can be seen from the EDS mapping results that Ti elements were evenly dispersed on the graphite. The XRD pattern shows a sharp peak at 2θ = 54.1°, corresponding to the (004) crystal of graphite and four weak diffraction peaks at 2θ = 35.9°, 41.8°, 60.5° and 76.4°, corresponding to the (111), (200), (220) and (222) crystal planes of TiC, which indicates that the TiC coating has successfully formed on the graphite.

**Figure 3:**
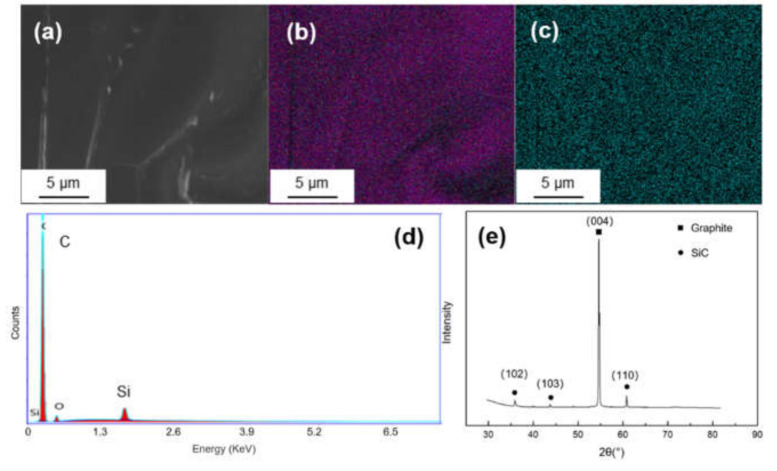
(**a**) SEM morphology and of Ti-coated graphite; (**b**,**c**) EDS mapping results of C and Si; (**d**,**e**) EDS spectrum and XRD pattern of the Si-coated graphite.

Figure 3 shows the SEM morphology and EDS mapping result of Si-coated graphite. It can be seen from Figure 3c that there is no obvious segregation of silicon on graphite. The EDS spectrum shows only Si and C signals. In the XRD pattern, there are three diffraction peaks at 2θ = 35.7°, 43.2° and 60.2°, corresponding to the (102), (103) and (110) crystal planes of SiC.

### 3.2. Microstructure Characterization of Ti-Coated Graphite/Al Composites

**Figure 4:**
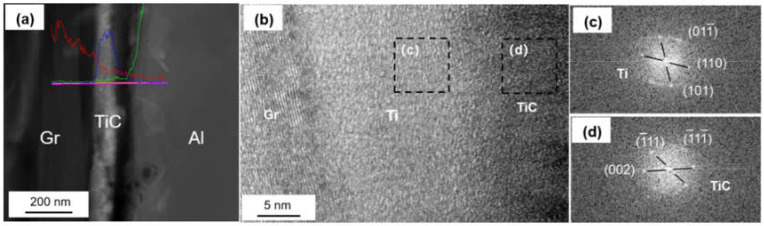
(**a**) Interface morphology of the Ti-coated Graphite/Al prepared by focused ion beam (FIB); (**b**) high resolution transmission electron microscope (HRTEM) image of Ti-coated graphite surface; (**c**,**d**) fast Fourier transform (FFT) of (**b**).

**Figure 5:**
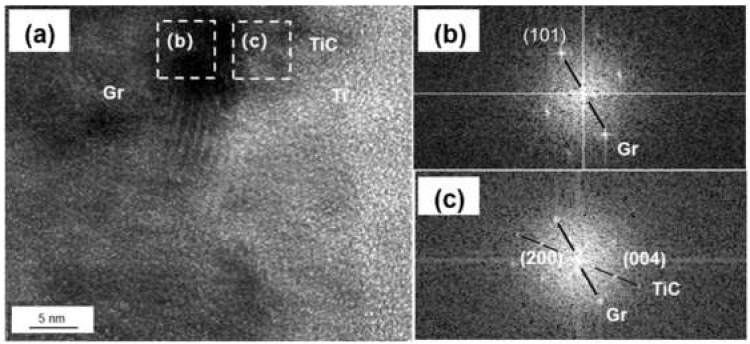
(**a**) HRTEM image of Ti-graphite surface; (**b**,**c**) FFT of (**a**).

The coated graphite and Al foils was fabricated into Graphite/Al composite, and the TEM samples containing the interface were prepared by focused ion beam (FIB). As shown in Figure 4a, continuous and uniform coating can be observed at the section, and the thickness of the coating was measured to be 183 nm. In order to further determine the phase and lattice orientation of the interface, the sample was observed by high resolution transmission electron microscope (HRTEM), and fast Fourier transform (FFT) was carried out to analyze the nanostructure with a digital micrograph. It can be noted from Figure 4b that the coating layer consisted of two different regions, including a thin, predeposited titanium layer with thickness of 15–20 nm; the rest of the area could be identified as a cubic structured TiC layer. In addition, nano-scaled TiC particles could be observed in partial interface areas between Ti and graphite (as shown in Figure 5). We speculated that TiC may be produced by the interfacial reaction under high temperature and high pressure, and the interface combination was enhanced to a certain extent.

**Figure 6:**
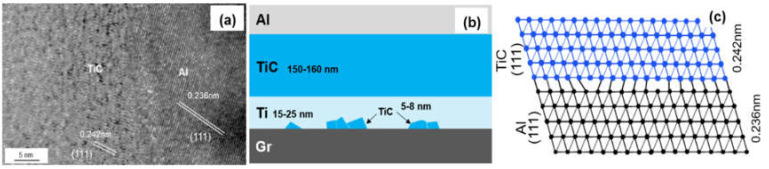
(**a**) HRTEM image of the TiC/Al interface; (**b**) Schematic drawing of interfacial structure of the Graphite/Al (Ti) composites; (**c**) Schematic diagram of the TiC/Al interfacial structure.

Figure 6a shows the HRTEM image of interfacial microstructure between TiC and Al, and the same lattice direction between (111)_TiC_ (0.242 nm) and (111)_Al_ (0.236 nm) can be observed. The transmission electron beam is parallel to the crystallographic orientation of [11]_TiC_ and [11]_Al_, respectively. The lattice mismatch can be calculated by the formula:

(4) $$\delta =\frac{2\left(d_{\mathrm{TiC}}-d_{\mathrm{Al}}\right)}{d_{\mathrm{TiC}}+d_{\mathrm{Al}}}\times 100\%$$

The mismatch between (111)_TiC_ and (111)_Al_ is 3.2%. Small mismatch means a good bonding strength between Al and Ti-coated graphite.

### 3.3. Microstructure Characterization of Si-Coated Graphite/Al Composites

**Figure 7:**
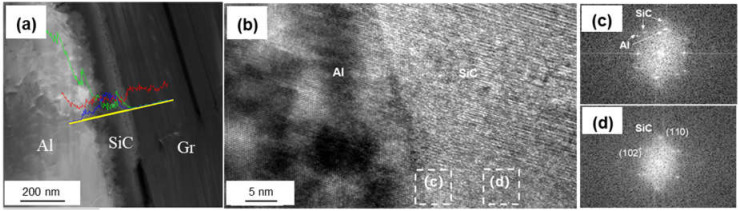
(**a**) Interface morphology of the Si-coated Graphite/Al prepared by FIB; (**b**) HRTEM image of SiC/Al surface; (**c**,**d**) FFT of (**b**).

**Figure 8:**
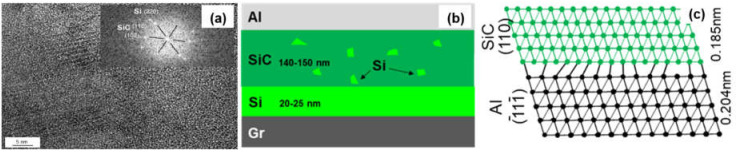
(**a**) HRTEM image of the SiC/Al interface; (**b**) Schematic drawing of interfacial structure of the Graphite/Al (Si) composites; (**c**) Schematic diagram of the SiC/Al interfacial structure.

Figure 7 shows the interfacial structure of the Si-coated Graphite/Al composites. The thickness of the coating layer was measured to be 167 nm. Al and SiC, with face-centered cubic structure, could be observed in the HRTEM image, and the interface between Al and the coating layer was very clear. From the FTT image, we can infer that the transmission electron beam is parallel to the direction of [110]_Al_ and [200]_SiC_, respectively. The crystal plane spacing between Al and SiC is quite different; thus, the (110)_Al_ does not grow along the (110)_SiC_, and a parallel lattice direction between (110)_SiC_ (0.185 nm) and ($\bar{1}$1$\bar{1}$)_Al_ (0.204 nm) could be recognized. Additionally, unreacted hexagonal silicon particles were observed in the coating layer (as shown in Figure 8). The mismatch between (110)_SiC_ and ($\bar{1}$1$\bar{1}$)_Al_ is 11.8%, which is equivalent to seven Al atomic lattice corresponding to eight SiC atomic lattice. This is because the space lattice of Al and SiC cannot match completely, and the bond energy of Al is larger than that of SiC. The formation of vacancy is beneficial to reduce the free energy of the interface and obtain a relatively stable interface bonding.

### 3.4. Thermal and Mechanical Properties

**Figure 9:**
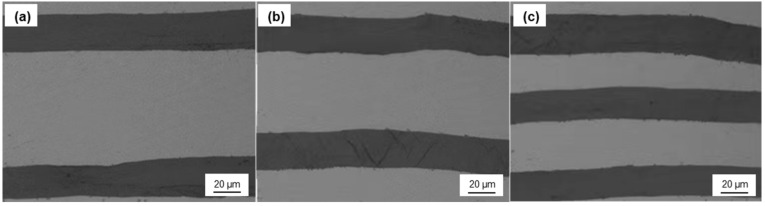
Optical microphotograph of the composite with different graphite volume fraction: (**a**) 11.1%; (**b**) 19.8%; (**c**) 28.7%.

In order to study the effect of graphite with different coatings (TiC, SiC, uncoated) on TC, the composites with different graphite volume fraction (11.1–28.7%) were fabricated by HIP. Figure 9 shows that the composites have an expected layered structure, and the graphite film and Al layer are arranged alternately. The orientation of graphite film was well-controlled, which is basically along the in-plane direction of the composite. The interface between graphite film and Al matrix was well-bonded, and there are no obvious holes and defects. This makes the orientation degree of the graphite film much better than that of the Graphite flake/Al composite, and this structure can give full play to the strengthening effect of high TC graphite film.

**Figure 10:**
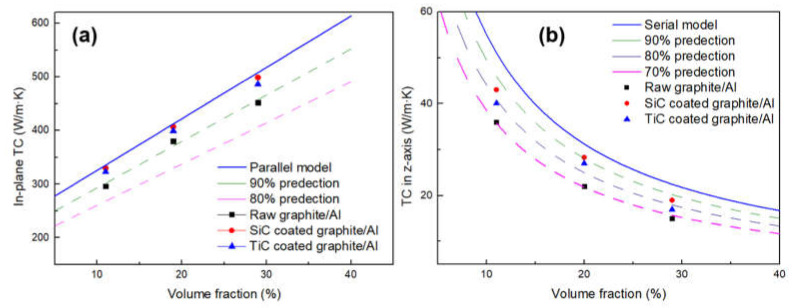
Comparison of the experimental TC of the composites to the predictions by TC model of laminate composites: (**a**) in-plane direction; (**b**) out-of-plane direction.

Figure 10 depicts the in-plane and out-of-plane TC of Graphite film/Al composites with different graphite volume fraction; the TC predictions of laminate composites were given by [18]:

In-plane direction:

(5) $$K=fK_{c}+\left(1-f\right)K_{m}$$

Out-of-plane direction:

(6) $$\frac{1}{K}=\frac{f}{K_{c}}+\frac{1-f}{K_{m}}$$

Here, f represents the volume fraction of graphite ands *K*,$\;K_{c}$ and $K_{m}$ represent the TC of composite, graphite film and Al, respectively.

It can be seen from Figure 10 that the carbide coating and increasing the volume fraction of graphite will both increase the TC of composites. After surface modification, the in-plane TC values of the composites with different volume fractions are all exceeding the 90% of the predictions, which could be due to the strong interfacial bonding and well-controlled orientation of graphite films. With increasing volume fraction of SiC-coated graphite from 11.1 to 28.7%, the in-plane TC increases from 330 to 499 W/m·K. As for TiC-coated graphite, the in-plane TC increases from 323 to 486 W/m·K. Compared with the composite reinforced by uncoated graphite, the TC of the composite is increased by 10.2% and 7.5% respectively. However, the TC in the z-axis shows a downward trend as the volume fraction of graphite increases. When the composite was reinforced by uncoated graphite, the TC was only 70% of the predictions, while this value can reach about 80% after carbide coating. This phenomenon can be attributed to the following reasons: the TC of graphite in the vertical direction is much lower than that in the plane direction, and the heat flow will be preferentially conducted in the direction of high TC, resulting in adverse effects on the vertical TC. In addition, when the heat flows along the vertical direction, it will pass through many interfaces; therefore, the TC is very sensitive to interfacial status, especially for the defects that may form in composites. To summarize, after the interface modification, the TC of the composites in the plane direction is very close to the theoretical value, which indicates that the carbide coating plays a positive role in improving the TC.

The heat conduction mode of graphite is phonon conduction, but the effect of phonon conduction in Al is very poor. The addition of carbide coating helps to alleviate the difference of phonon conduction between graphite and Al. Therefore, it is necessary to further study the effect of different carbide coating on the interfacial thermal conductance (ITC) of the composite. The TC in the out-of-plane direction was selected as the research object in order to consider the effect of carbide coating. It can be solved by replacing the measured TC value with an effective TC, including the effect of ITC,$\;K^{eff}$, which was given by [19]:

(7) $$K^{eff}=\frac{K_{c}}{1+\frac{2K_{c}}{hD}}$$

Here, $K_{c}\;\mathrm{is}$ the intrinsic TC of graphite, *h* is the ITC and D is the diameter of the inclusion. Thus, the actual ITC can be calculated by Equation (6), while the theoretical ITC could be estimated by the acoustic mismatch model (AMM) [20]:

(8) $$h^{t}≅\frac{1}{2}\rho_{m}c_{m}\frac{v_{m}^{3}}{v_{c}^{2}}\frac{\rho_{m}v_{m}\rho_{c}v_{c}}{{\left(\rho_{m}v_{m}+\rho_{c}v_{c}\right)}^{2}}$$

where ρ, *c* and *v* are the mass density, the specific heat capacity and the phonon velocity, respectively. The subscripts c and m refer to the graphite and metal matrix, respectively. The relevant parameters of the materials used for the calculation are tabulated in Table 2.

The theoretical ITC between graphite and Al was calculated to be 4.3 × 10^7^ W/m^2^ K. The carbide coating can act as the intermediate layer for phonon propagation, which forms an effective phonon velocity gradient at the interface of the composite, so that the phonon scattering at the interface can be suppressed. After introducing carbide coating, the theoretical ITC of SiC-coated graphite and TiC-coated graphite was increased to 6.7 × 10^7^ W/m^2^ K and 5.5 × 10^7^ W/m^2^ K, respectively.

**Figure 11:**
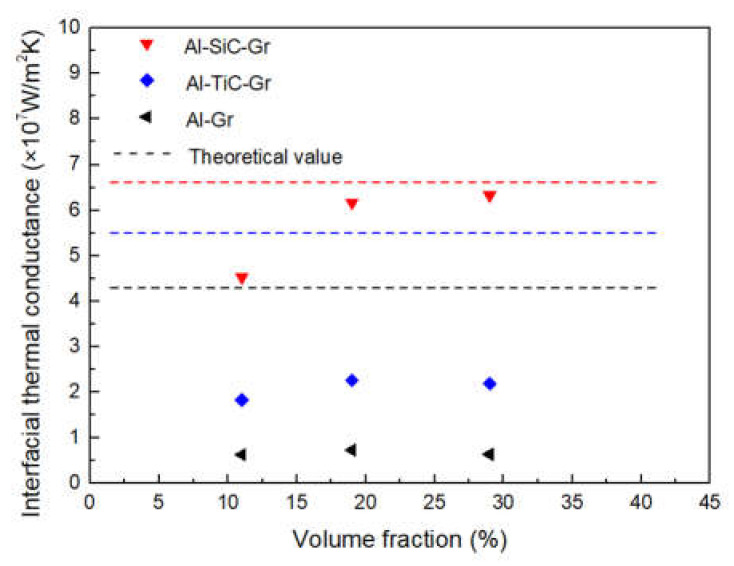
Comparison of interfacial thermal conductance to different composites.

As shown in Figure 11, the actual ITC calculated by Equation (6) is in the same order of magnitude as the theoretical value. In this study, the thickness of the coating layer is very thin, so the theoretical ITC values of TiC and SiC are very close. However, it should be noted that, due to the low TC of TiC, the ITC will decrease with the increase of thickness. From the calculated value of the experiment, there is still a certain gap between the experimental value and the theoretical value, especially for the uncoated graphite, which may be due to the interface defects caused by the poor wettability between aluminum and graphite. Through the surface modification, the ITC of the composites has been improved, correspondingly, but the interface reaction between TiC coating and graphite may aggravate the phonon scattering at the interface, so the improvement of ITC is relatively constrained. Compared with TiC coating, the interface of SiC is relatively pure and firmly bonded with the graphite and metal matrix, which can more effectively improve the ITC.

**Figure 12:**
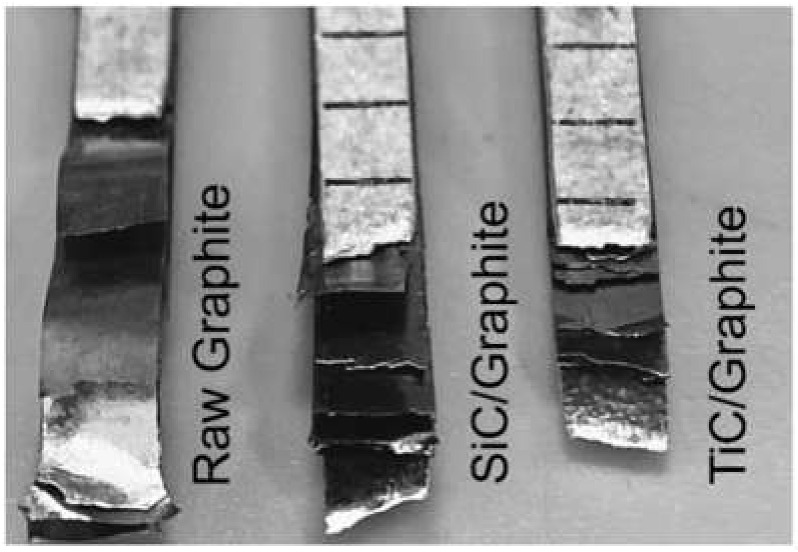
Tensile fracture morphology of composites.

The mechanical properties of composites with different volume fractions of graphite are listed in Table 3. Obviously, the tensile strength sharply decreases with the increasing of the volume fraction of graphite, and the mechanical properties of the composites become very sensitive to the interface bonding when the volume fraction of graphite is high. The experimental measurements for coated Graphite/Al composites show higher values than that of the raw Graphite/Al composites. As is shown in Figure 12, owing to the weak bonding between raw graphite and metal matrix, the graphite was completely disengaged and pulled out from matrix. For 28.7 vol.% raw Graphite/Al composite, the tensile strength is 33 MPa. When the graphite is coated with SiC or TiC, the tensile strength increased to 39 MPa and 44 MPa, respectively. Additionally, the fracture of composite reinforced by coated graphite is very even, and the graphite remains bonded with matrix. Compared with SiC coating, TiC-coated graphite has higher mechanical properties, which may be due to the lower crystal plane mismatch between TiC and matrix and the interface reaction with graphite, which both improve the mechanical properties.

## 4. Conclusions

The carbide-coated graphite and the interface of the Graphite/Al composites were systematically studied to reveal their influence on thermal and mechanical properties. By controlling the deposition interfacial structure, continuous and uniform carbide coating with thickness less than 200 nm was successfully deposited on the graphite surface. The nanometer-scale carbide coating improved the bonding between graphite and Al, the phonon scattering at the interface was reduced accordingly and the ITC of SiC@Graphite/Al and TiC@Graphite/Al composites were increased by 9.8 times and 3.4 times, respectively. After surface modification, the in-plane TC of the composites with different volume fractions were all exceeding the 90% of the predictions, with increasing volume fraction of graphite from 11.1 to 28.7%. The TC of SiC@Graphite/Al composites increased from 330 to 499 W/m·K; for TiC@Graphite/Al composites, the TC increased from 323 to 486 W/m·K, which was 10.4% and 7.5% higher compared to that of the Graphite/Al, respectively. In addition, the mechanical properties of the composites reinforced by carbide-coated graphite were also improved.

## Figures and Tables

**Table 1:** Crystallite parameter of graphite film.

Parameter	2θ (°)	FHWM	*d*_002_ (nm)	g (%)
Value	26.549	0.225	0.3357	96.51

**Table 2:** Parameters of materials for the acoustic mismatch model (AMM) model.

Material	TC (W/m·K)	Specific Heat (J/kg·K)	Phonon Velocity (m/s)	Density (kg/m^3^)	Reference
Graphite	1189	710	14,800	2100	[11]
Aluminum	230	904	12,164	2700	[21]
TiC	17	568	6777	4930	[22]
SiC	483	670	7925	3220	[23]

**Table 3:** Mechanical properties of composites with different volume fractions of graphite.

Surface Condition of Graphite	Volume Fraction (%)	Tensile Strength (MPa)	Yield Strength (MPa)	Elongation Rate (%)
Raw graphite	11.1	106	50	22.5
	19.8	68	29	10.3
	28.7	32	-	4.3
TiC-coated	11.1	114	58	25.5
	19.8	82	43	11.4
	28.7	44	23	6.7
SiC-coated	11.1	112	58	25.5
	19.8	74	35	10.4
	28.7	39	19	5.6

## Data Availability

Data is contained within the article.
